# Effects of Freeze–Thaw Cycles on Water Migration, Microstructure and Protein Oxidation in Cuttlefish

**DOI:** 10.3390/foods10112576

**Published:** 2021-10-26

**Authors:** Ying Lv, Jing Xie

**Affiliations:** 1 College of Food Science and Technology, Shanghai Ocean University, Shanghai 201306, China; 15900328167@163.com; 2 Shanghai Engineering Research Center of Aquatic Product Processing & Preservation, Shanghai Ocean University, Shanghai 201306, China; 3 National Experimental Teaching Demonstration Center for Food Science and Engineering, Shanghai Ocean University, Shanghai 201306, China; 4 Collaborative Innovation Center of Seafood Deep Processing, Ministry of Education, Dalian 116034, China

**Keywords:** cuttlefish, protein oxidation, quality, multiple freeze–thaw cycles

## Abstract

This study was conducted to analyze the effects of multiple freeze–thaw (F-T) cycles on microstructural disruption, water migration, protein oxidation and textural properties of cuttlefish. Low-field nuclear magnetic resonance (LF-NMR) showed an increase in the proportion of free water in cuttlefish flesh. It was also observed by scanning electron microscopy (SEM) that multiple F-T cycles increased the gap between muscle fibers and disrupted the original intact and compact structure. The results of Fourier transform infrared spectroscopy, intrinsic fluorescence spectroscopy, Ca^2+^ATPase content, sulfhydryl content and free amino acid content indirectly prove that multiple F-T cycles can lead to the destruction of the a-helical structure of cuttlefish myofibril protein and the content of irregular curls increased, protein aggregation and degradation, and tryptophan oxidation. In addition, after repeated freezing and thawing, the water holding capacity, whiteness value, elasticity and chewiness of cuttlefish flesh decreased, the total volatile base nitrogen content increased. It can be concluded that the freeze–thaw cycles are very harmful to the quality of the frozen foods, so it is important to keep the temperature stable in the low-temperature food logistics.

## 1. Introduction

Cuttlefish is a marine mollusk, belonging to cephalopods. It is widely distributed along China’s coast, especially in the Zhejiang Province, where it produces the most [1]. It is one of the four major kinds of seafood in China (big yellowtail, small yellowtail, scallop and cuttlefish), and the fishery catch is very large. The cuttlefish has a high nutritional value and is rich in medicinal value. In addition to being rich in protein and the amino acids required by the human body, squid also contains a large amount of taurine, which can inhibit the cholesterol content in the blood, relieve fatigue, restore vision and improve liver function. Rich in calcium, phosphorus and iron, it is good for bone development and blood production and can effectively treat anemia, and the selenium it contains has anti-viral and anti-radiation effects.

It is known that aquatic products contain proteins, active peptides, unsaturated fatty acids and other minerals, and they contain their own endogenous autolytic enzymes with high activity [2]. Therefore, if there is no proper low-temperature storage conditions, it is prone to spoilage and deterioration, thus affecting the quality of fish [3,4,5]. Freezing plays a major role in ensuring the distribution of meat products supplied to the world [6,7]. However, the freezing process produces ice crystals of varying sizes, which cause some mechanical damage to cell membranes and tissue structures, resulting in a reduction in quality [8]. The thawing process can also result in nutrient loss and reduction of soluble protein content. Repeated freezing and thawing makes the ice crystals in the food recrystallize, resulting in the destruction of cell structure, protein content decline, fat oxidation, color deterioration, and juice loss, thus reducing the edible value of aquatic and meat foods. Because the current cold chain technology is not perfect, especially in the transportation, and temperature fluctuations inevitably occur, this results in a repeated freezing and thawing process. It contributes to the changes in the physical and chemical properties of the muscle and results in its reduced quality and economic losses [9]. In recent years, much headway has been made in the assessment of fish freshness and safety, but little research has been undertaken on cephalopods, much of it on squid [2,10]. The effects of multiple F-T cycles on muscle protein and quality have been receiving attention from several scholars both at home and abroad [11,12]. At present, few studies on the effect of multiple F-T cycles on the quality of cuttlefish have been reported.

Therefore, using cuttlefish as research objects, protein content, fat oxidation, total volatile base nitrogen (TVB-N), and thawing juice loss rate as evaluation indexes, combined with texture profile analysis, the effect of repeated freezing–thawing on and quality changes of cuttlefish was proposed and used to provide a theoretical basis for transportation, storage, processing, and marketing in actual production.

## 2. Materials and Methods

### 2.1. Sample Preparation

Fresh cuttlefish from the same lot (dead for no more than two days) weighing 1000 ± 100 g were purchased from Luchaogang, Pudong New Area, Shanghai, China. Fresh cuttlefish were taken as the control group for 0 freeze–thaws. The sample was frozen in a refrigerator at −23 °C for 24 h and then thawed in a refrigerator at 4 °C until the core temperature of the sample was 2 °C, measured with a thermometer inserted into the center of the cuttlefish, and the above operation was considered as 1 freeze-thaw. The experiments were carried out for 0, 1, 2, 3, 4 and 5 freeze–thaw treatments, and the samples from each treatment were subjected to experimental analysis.

### 2.2. Thawing Loss

Thawing loss was measured by Tan et al. [13]. The weight of samples was measured before freezing (M1) and after freeze–thaw cycle (M2), and then Formula (1) was used for the calculation of thawing loss:

(1) $$\mathrm{Thawing}\;\mathrm{Loss}/\%=\frac{M2}{M1}\times 100\%$$

### 2.3. Cooking Loss

Cooking loss was extracted according to the method of Lan et al. [14]. The thawed cuttlefish flesh was divided into small pieces of 2 × 2 × 1cm. Weighed before steaming (M3), placed in a sealed bag, heated in a water bath at 85 °C for 20 min, then removed and cooled to room temperature, the water on the surface of the cuttlefish flesh pieces was absorbed with paper and weighed after steaming (M4). The cooking loss rate of cuttlefish was calculated according to Equation (2).

(2) $$\mathrm{Cooking}\;\mathrm{Loss}/\%=\frac{M3-M4}{M3}\times 100\%$$

### 2.4. Centrifugal Loss

The centrifugal loss was measured by Tan et al. [15]. Remove about 2 g of thawed cuttlefish flesh and record its weight accurately as M5. Wrap it with filter paper and place it in a centrifuge tube. Centrifuge at 5000 rpm for 10 min at 4 °C. At the end of centrifugation, it was removed and weighed and recorded as M6. The centrifugal loss was calculated according to Equation (3).

(3) $$\mathrm{Centrifugal}\;\mathrm{loss}\left(\%\right)=\frac{M_{5}-M_{6}}{M_{5}}\times 100\%$$

### 2.5. Low-Field Nuclear Magnetic Resonance(LF-NMR) and Proton Magnetic Resonance Imaging (MRI)

LF-NMR and MRI extracted according to the procedure used by Lan et al. [16]. T2 measurements were performed using a MesoMR23-060H.I LF-NMR analyzer (Niumag Corporation, Shanghai, China). The parameters were set according to the method of Lv et al. [17]. Imaging resonance images were made using the PQ001 benchtop pulsed MRI analyzer, which also performed pseudo-colorization.

### 2.6. Color Properties Analysis

The color values L*, a* and b* of the flesh on the inside of the thawed cuttlefish were measured by a CR-400 colorimeter (Konica Minolta, Tokyo, Japan), and whiteboard correction was performed before the measurement of the cuttlefish products.

### 2.7. pH

pH value was extracted according to the method of Song et al. [18]. Measured using a pH meter (Sartorius, Gottingen, Germany). Dilute 2 g of cuttlefish with distilled water to form a 20 mL homogenate. Use a pH meter (Sartorius, Gottingen, Germany) to measure the pH.

### 2.8. Texture Profile Analysis (TPA)

The texture properties of cuttlefish flesh were measured according to the method of Tan et al. [19]. Thawed cuttlefish meat thawed to 4 °C was removed, cut into 4 × 4 × 1 cm squares and measured using a texture analyzer (TMS-Pro, FTC Corporation, Washington, DC, USA) with a P/6 flat-bottom column probe.

### 2.9. Myofibrillar Proteins (MP) Extraction

MP was extracted according to the method of Lv et al. [17]. A total of 2 g of cuttlefish flesh and 20 mL 20 mmol/L Tris-maleate (0.05 mol/L KCl, pH = 7.0) were mixed, homogenized and then centrifuged at 10,000 rpm for 10 min, and the supernatant was discarded, and the above process was repeated twice. To the obtained precipitate, 20 mL of 20 mmol/L Tris-maleate (0.6 mol/L KCl, pH = 7.0) was added, homogenized and then extracted at 4 °C for 3 h, centrifuged at 10,000 rpm for 10 min, and the supernatant was myofibrillar protein solution.

### 2.10. Total Sulfhydryl (SH) Group Content and Ca^2+^-ATPase activity

The content of total sulfhydryl groups and the activity of Ca^2+^-ATPase were determined using the methods described in A063-1 (Jiancheng, Nanjing, China) and A070-4 (Jiancheng, Nanjing, China), respectively [20]. The reagents were added to the 10% homogenate supernatant sequentially according to the method attached to the kit, and the absorbance was measured at 412 and 636 nm using an enzyme marker (Thermo Scientific, Shanghai, China), respectively, after the reaction was completed.

### 2.11. Free Amino Acids (FAA)

Refer to Wang et al. [21] for the determination method. A total of 2.0 g of cuttlefish meat was homogenized with 10 mL of 5% trichloroacetic acid (TCA) and then centrifuged at 8000 rpm for 10 min. The residue was extracted twice with the same volume of 5% TCA. The supernatant was collected in a volumetric flask and diluted to 25 mL. FAAs and ammonia were analyzed in an L-8900 amino acid analyzer (Hitachi High-Tech Co., Ltd., Tokyo, Japan) using 10 µL of the diluted solution.

### 2.12. Fourier Infrared Spectroscopy

The secondary structure of cuttlefish proteins was studied by freeze-drying pre-extracted cuttlefish myofibrillar proteins for 72 h, with slight modifications referring to the method of Wang et al. [20]. Using an FT-IR spectrometer (Spotlight 400, PerkinElmer Instruments, Waltham, MA, USA), approximately 0.2 g of lyophilized MP was spread on the surface of attenuated total reflection crystals with a wavelength range set to 600–4000 cm^−1^, and the spectra were collected.

### 2.13. Intrinsic Fluorescence Spectra

According to Chu’s method [22], intrinsic fluorescence spectra were measured using the F-7100 fluorescence spectrophotometer (Hitachi Co., Tokyo, Japan). The protein solutions were excited at 290 nm (slit width was 5 nm), and the emission spectra were recorded from 300 to 410 nm at a scanning speed of 1200 nm/min.

### 2.14. Total Volatile Base Nitrogen (TVB-N)

Determination of TVB-N by the method of Liu et al. [23]. A total of 5 g of minced cuttlefish flesh was weighed and measured with the automated Kjeltec nitrogen analyzer (Kjeltec 8400, Foss, Denmark).

### 2.15. Determination of Microstructure by SEM

Refer to the method of Lv et al. [17].

### 2.16. Statistical Analysis

Each group was set up into three parallel groups, and a total of 18 fresh cuttlefish were used in the experiment. The experiment used SPSS 20.0 for statistical analysis. The values in the article are expressed as mean ± standard deviation difference, and significant differences between means were assessed by Tukey’s (HSD) comparison test with a significance level of *p* < 0.05. Plots were made using Origin 2021 software.

## 3. Results and Discussion

### 3.1. Water Holding Capacity

**Figure 1:**
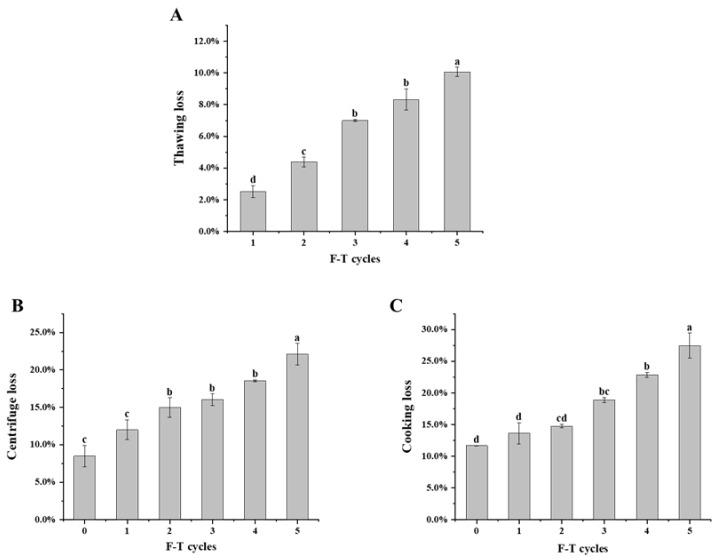
Thawing loss (**A**), centrifugal loss (**B**) and cooking loss (**C**) of cuttlefish after F-T cycles. Error bars show standard deviation. The letters “a–d” indicate significant differences (*p* < 0.05).

Figure 1A shows the effect of F-T cycles on the change of the thawing loss rate of cuttlefish. The results showed that the thawing losses increased to 2.53%, 4.39%, 7.0%, 8.32% and 10.07% after the 1st, 2nd, 3rd, 4th and 5th freeze–thaw cycles. Figure 1B,C shows that the cooking loss and centrifugal loss had the same tendency as the thawing loss, and all the three indexes showed an elevated trend with increasing number of F-T cycles (*p <* 0.05). The centrifugal and cooking losses of fresh cuttlefish were 8.5% and 11.66%, respectively. For centrifugal loss and cooking loss, there is no significant difference between one cycle and zero cycles (*p <* 0.05), while they reached 22.11% and 27.46% after five freeze–thaw cycles, with an increase of 13.61 and 15.8 percentage points, respectively. When aquatic products were frozen, the fluid in the body formed ice crystals of varying sizes. These ice crystals caused mechanical damage to the cells, and the solution inside the cells then flowed out during thawing if it could not be absorbed. When aquatic products were repeatedly frozen and thawed, the ice crystals in the body recrystallized and the damage to the cell membrane was even more serious [6].

### 3.2. Moisture Distribution

**Figure 2:**
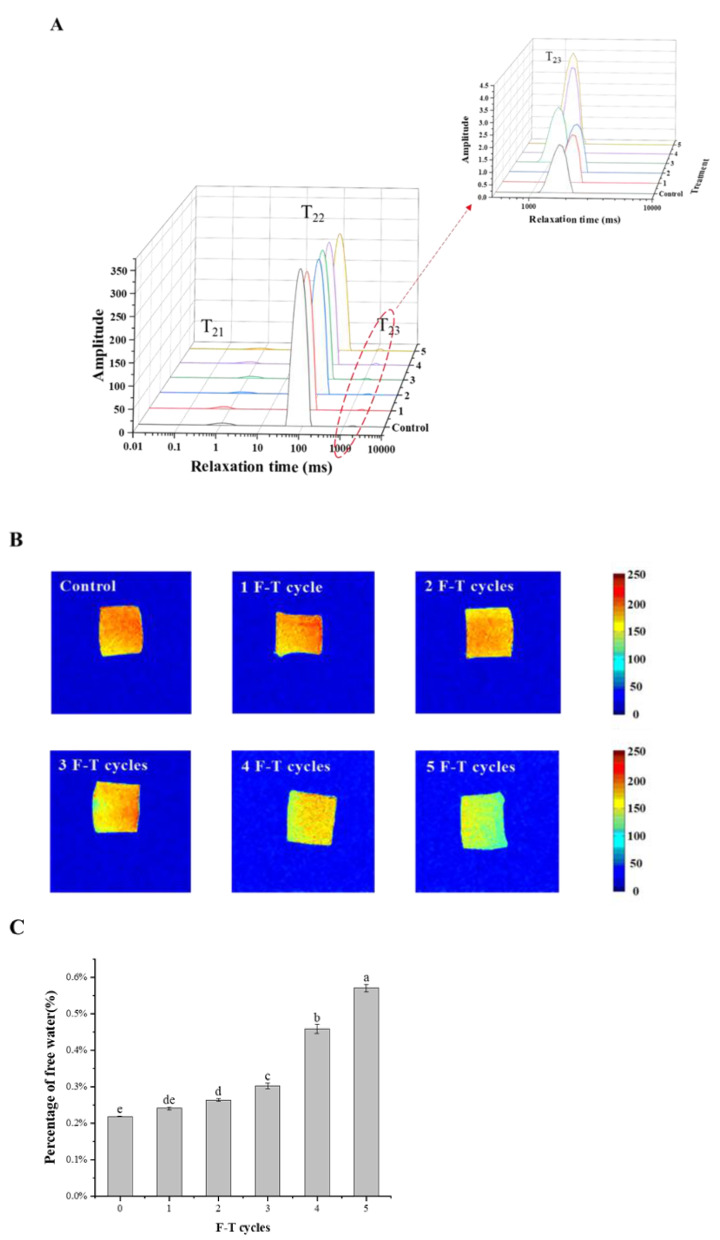
T2 relaxation time distribution of LF-NMR (**A**), magnetic resonance imaging (**B**), percentage of free water content (**C**) of cuttlefish after F-T cycles. Error bars show standard deviation. The letters “a–e” indicate significant differences (*p* < 0.05).

Figure 2A shows the three-dimensional plot of the variation of the transverse relaxation time for the six groups of samples, and it can be seen that there are three peaks in the distribution of T2 within the relaxation time of 0–10,000 ms. T21 represents bound water, which is mainly bound to proteins and other macromolecules. T22 represents immobile water, which is the most abundant form of water in meat, taking up more than 98% of the total water content of cuttlefish, and T23 represents free water present in the extracellular space.

It can be seen from Figure 2B that the content of free water increased significantly (*p* < 0.05) with the increase in the number of F-T cycles. The free water content of cuttlefish meat after zero F-T cycles was 0.219%, and there was no significant difference between one freeze–thaw cycle and zero. After five F-T cycles, the content of free water increased to 0.571%. This was due to the physical destruction of cuttlefish muscle cells by ice crystals during the F-T process and the weakening of the binding force between water and protein molecules due to protein oxidation, which caused an increase in the free water content [24]. Therefore, the control samples showed lower juice loss and poorer water holding capacity [25].

The red color in the pseudo-color map of nuclear magnetic imaging indicates 1H high proton density, and the high proton density represents the high water content of that part of the fish, and the blue color indicates low water content, so the water content and migration characteristics can be reflected by the signal color change [26]. As can be seen in Figure 2C, after three freeze–thaw cycles, a distinct blue color appeared at the edges of the flesh, and by the fifth freeze–thaw cycle, a large blue area appeared. The increase in blue areas indicates more juice loss, which is consistent with the conclusion of water holding capacity in the above reaction.

### 3.3. Colour

As can be seen from Table 1, there was no significant difference in L*, a*, b* of cuttlefish meat between zero F-T cycles and one F-T cycle (*p* < 0.05). The L* of cuttlefish in the first three freeze–thaw cycles showed a remarkable decreasing trend (*p* < 0.05), which may be due to protein and lipid oxidation. However, after the fourth time, it increased significantly, which may be due to the continuous recrystallization of ice crystals in the three freeze–thaw cycles. The thawing process destroyed the integrity of the fish tissues, resulting in the increase in free water content between tissues, the increase in light reflectivity and brightness on the surface of cuttlefish flesh.

### 3.4. pH

**Figure 3:**
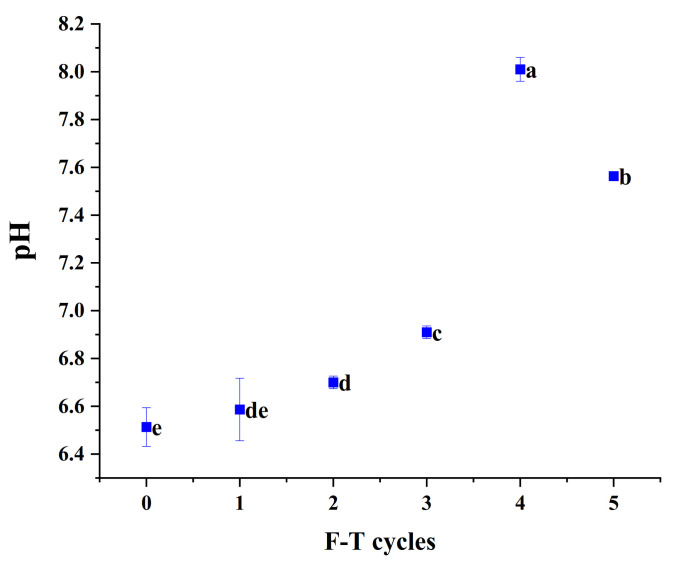
The pH value of cuttlefish after freeze–thaw cycles. Error bars show standard deviation. The letters “a–e” indicate significant differences (*p* < 0.05).

As shown in Figure 3, the pH value of fresh cuttlefish flesh was 6.51. The pH of cuttlefish flesh showed an increasing trend (*p* < 0.05) during the first four freeze–thaw cycles. In particular, the pH reached 8.01 after four freeze–thaw cycles, which was 1.1 higher than the third time. The increase in pH may be due to the decomposition of proteins in the fish body into alkaline substances such as amines. The decrease after the fifth freeze–thaw may be due to the enzymatic decomposition of neutral fatty acids and phospholipids in the meat to produce free fatty acids. The enzymatic degradation of ATP occurred with the release of inorganic phosphate and ammonia, which was also associated with the change in pH value.

### 3.5. TPA

Table 2 shows the changes in hardness, elasticity, chewiness and cohesion of cuttlefish after F-T cycles. After five freeze–thaw cycles, the hardness of cuttlefish meat dropped to nearly a quarter of that of fresh cuttlefish flesh. Cuttlefish meat is rich in proteins, and the proteins and their hydrated layers form a mesh structure and thus have some resistance to external forces, and this resistance was expressed as the elasticity of the meat. The elasticity of cuttlefish meat did not differ significantly after the first three freeze–thaws compared to fresh samples (*p* < 0.05) and decreased significantly when freeze–thaws were performed four times (*p* < 0.05). The trend of cuttlefish cohesiveness was similar to elasticity. The masticatory properties of cuttlefish flesh decreased the most after the F-T cycle, and after the fifth F-T cycle, the masticatory properties of cuttlefish flesh were only 10,326.34, which was only one-third of the fresh sample. This may be due to the destruction of the natural structure by ice crystals in the cells during the repeated freeze–thaw treatment, resulting in poor resistance to external forces [27], leading to a decrease in the hardness, elasticity, and masticatory properties of cuttlefish flesh [28]. Repeated freeze–thawing can reduce the acceptability to consumers.

### 3.6. Total Sulfhydryl Content

**Figure 4:**
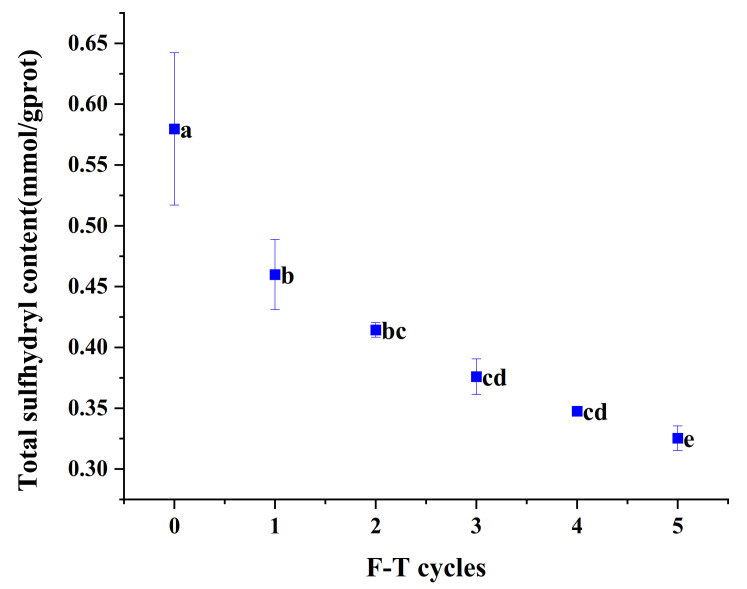
The total sulfhydryl content of cuttlefish after freeze–thaw cycles. Error bars show standard deviation. The letters “a–e” indicate significant differences (*p* < 0.05).

During freeze–thaw, changes in the structure of the head region of actin, which exposes the buried sulfhydryl groups inside the protein and oxidizes them into disulfide bonds, lead to a decrease in the content of sulfhydryl groups [29]. As can be seen from Figure 4, the total sulfhydryl content decreased with the increasing number of F-T cycles (*p* < 0.05). The content of total sulfhydryl groups in fresh cuttlefish flesh was 0.58 mmol/g prot, which was significantly reduced after one freeze–thaw cycle. The decrease in total sulfhydryl content was not marked (*p* < 0.05) in the first four freeze–thaw cycles, but there was a significant decrease (*p* < 0.05) in the fifth freeze–thaw cycle.

### 3.7. Ca^2+^-ATPase Activity

**Figure 5:**
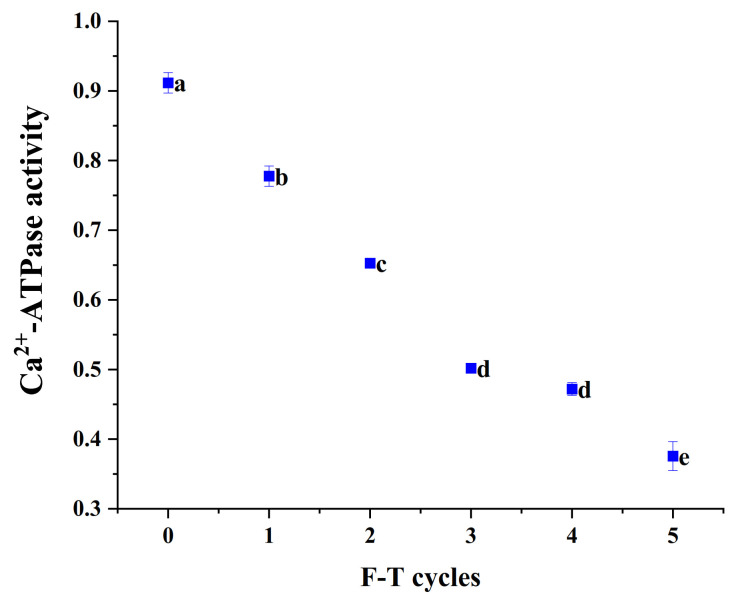
The Ca^2+^-ATPase activity of cuttlefish after freeze–thaw cycles. Error bars show standard deviation. The letters “a–e” indicate significant differences (*p* < 0.05).

Myosin heads are very sensitive to the freeze–thaw process, and their molecular conformation is prone to change, leading to a decrease in Ca^2+^-ATPase activity. The activity of Ca^2+^-ATPase is often considered to be indicative of the denaturation of myogenic fibronectin [30]. As can be seen from Figure 5, the number of freeze–thaws had a significant effect on Ca^2+^-ATPase activity of cuttlefish (*p* < 0.05). The initial Ca^2+^-ATPase activity of cuttlefish tissues was 0.91 U/mg prot, and the enzyme activity decreased with the increase in freeze–thaw cycles. The rate of decrease in Ca^2+^-ATPase activity slowed down after the third freeze–thaw cycle, the activity of Ca^2+^ATPase decreased to 0.38 U/mg prot. Protein interactions and aggregation in the freeze–thaw cycle had relation to the decrease in ATPase activity [31]. In addition, oxidation of sulfhydryl groups in the myosin head may also bring about a decrease in Ca^2+^-ATPase activity [32].

### 3.8. FAA

Free amino acids are also used as quality control indicators for kinds of aquatic products. As shown in Table 3, 17 free amino acids were detected from cuttlefish meat, among which the main amino acids were pro line, arginine and alanine. They accounted for about 39.76% of the total amino acids. The two biological amines that have the greatest impact on human health, histamine and tyramine, are formed by the direct decarboxylation of histidine and tyrosine, respectively. The content of these two amino acids is directly related to the corruption of cuttlefish [33]. After five freeze–thaw cycles, histidine and tyrosine increased from the initial 7.04 and 15.96 mg/100 g to 20.7 and 46.88 mg/100 g, respectively. This shows that as the number of freeze–thaw cycles increases, the corruption of cuttlefish deepens. Methionine and cysteine are sulfur-containing amino acids, both of which have a bad effect on the taste of fish meat. After five cycles, the content increased from 15.36 and 2.24 mg/100 g to and 3.44 mg/100 mg [34]. After the freeze–thaw cycle, the total free amino acids increased from the initial 363.945 to 813.187 mg/100 g. This is because proteolytic enzymes hydrolyze the protein in cuttlefish muscle and connective tissue during the freeze–thaw cycle [34,35].

### 3.9. Protein Secondary Structure

**Figure 6:**
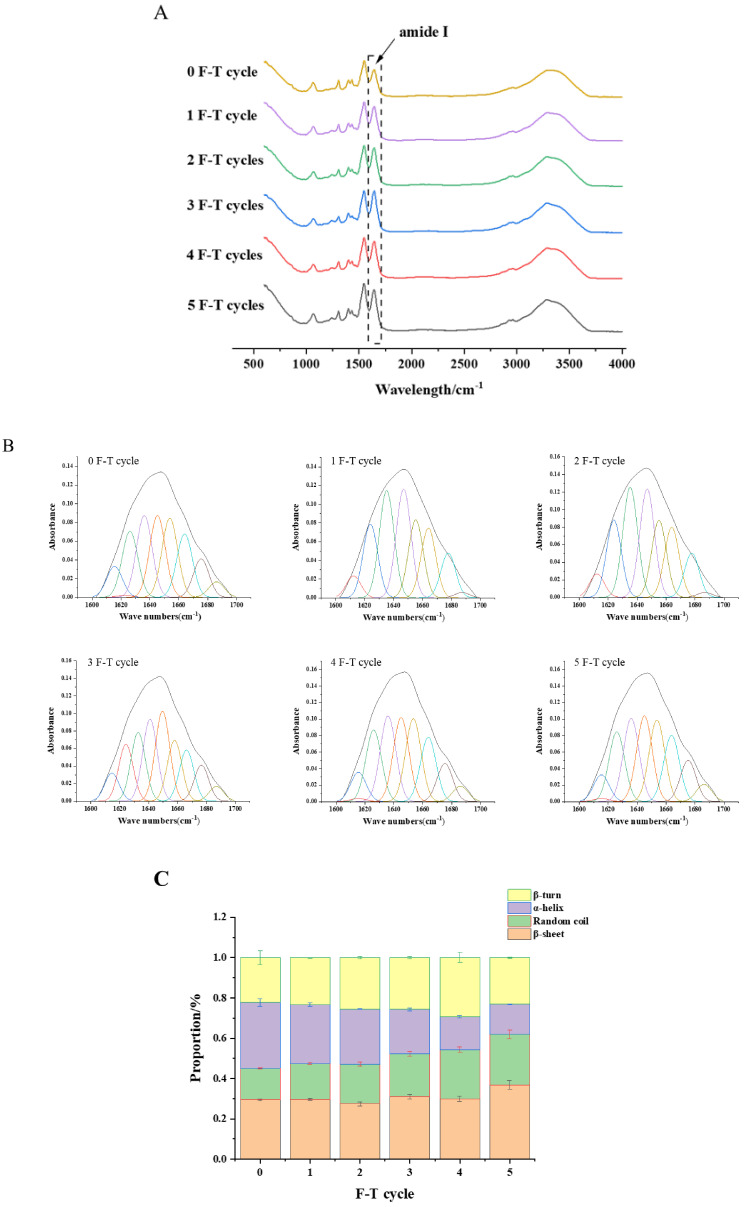
FTIR spectra (**A**), iterative curve-fitted (**B**), secondary structure content (**C**) of cuttlefish after F-T cycles. Error bars show standard deviation.

Figure 6A shows the infrared spectra of freeze-dried cuttlefish meat-like proteins scanned in the full band from 600 to 4000 cm^−1^. The characteristic absorption peaks in the figure are mainly caused by peptide and protein secondary structure vibrations. The absorption peaks in the 1700~1600 cm^−1^ band caused by the C=O stretching vibration are usually referred to as the amide I band, and the study of the protein secondary structure is analyzed in this band, and the vibration frequency of the amide I band component is closely linked with the secondary structure of each protein [29,36].

PeakFit EXE software was used to analyze the ATR-FTIR spectra in the 1700–1600 cm^−1^ band. After deconvolution, second-order derivation and curve fitting, the information of individual IR peaks is obtained, as shown in Figure 6B, and the content of the protein secondary structure is shown in Figure 6C. With the increase in the number of freeze–thawing, the α-helical content of cuttlefish gradually decreased, while the content of irregular curl gradually increased, and the trends of β-turn and β-folding were not obvious. The content of α-helix in the control group was 32.63%, and the content of α-helix decreased by 3.52%, 10.47%, and 17.72% after one, three, and five freeze–thaw cycles, respectively. The content of irregular curl in the control group was 15.64%, and the contents of irregular curl after one, three, and five freeze–thaw cycles were 17.89%, 21.15%, and 25.05%, which increased by 2.24%, 5.51%, and 9.41%, respectively. It indicates that the protein structure changes from regular to sparse [15]. During multiple freeze–thaw cycles, protein denaturation, sulfhydryl oxidation and weakening of the hydrogen bond between water and protein lead to the disruption of the protein spatial conformation [25,26,37].

### 3.10. Intrinsic Fluorescence Spectroscopy Analysis

**Figure 7:**
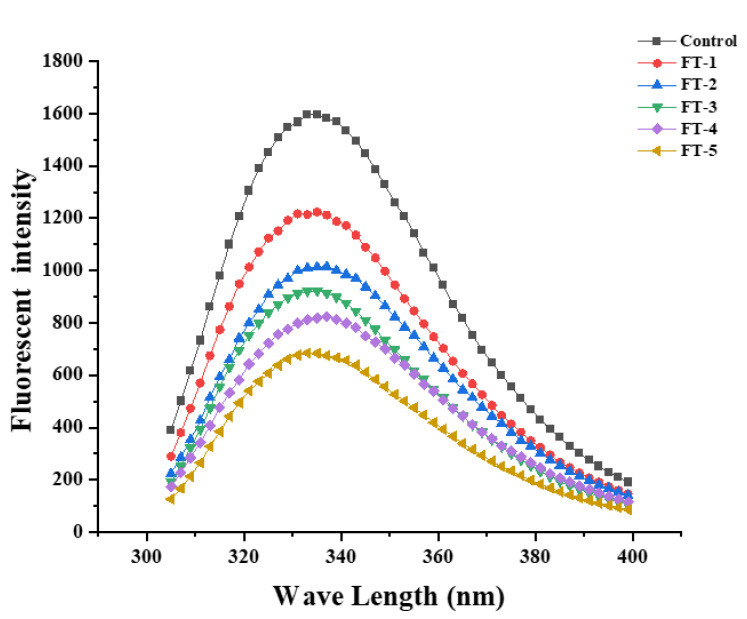
Intrinsic fluorescence emission spectra of MP of cuttlefish after freeze–thaw cycles.

As can be seen from Figure 7, the fluorescence intensity of myogenic fibronectin reduced significantly with the increasing cycles of freeze–thaw treatments, which indicated the unfolding of the protein tertiary structure. This is because the protein endogenous tryptophan fluorescence is very susceptive to the polarity of its surrounding microenvironment [38]. When the protein exists in a folded state, the tryptophan residues are mainly located in a hydrophobic environment such as the protein core, when the excited tryptophan has a relatively high fluorescence intensity [39]. If the protein is unfolded, tryptophan residues are more exposed on the surface of the protein molecule, and the fluorescence intensity of the excited tryptophan decreases. In addition, it is possible that free radicals and fat oxidation products such as hydrogen peroxide, MDA and ketones can oxidize tryptophan residues or bind them to other products, which can also lead to a decrease in protein fluorescence intensity [26].

### 3.11. TVB-N

**Figure 8:**
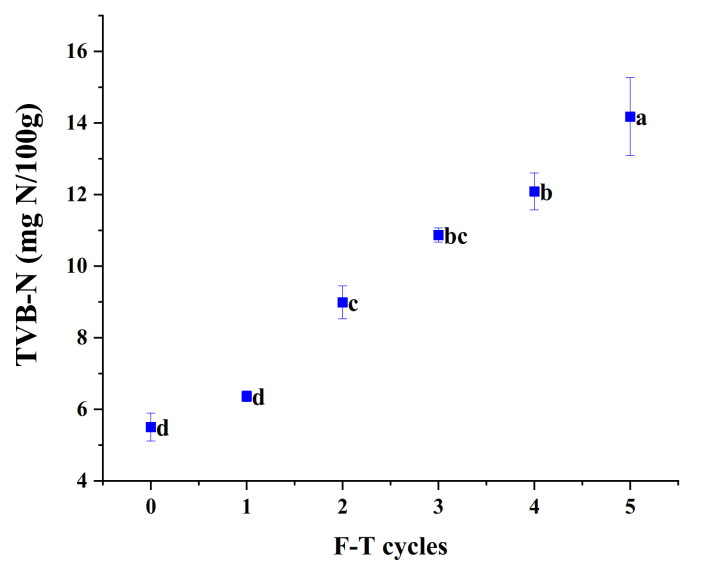
The content of TVB-N of cuttlefish after freeze–thaw cycles. Error bars show standard deviation. The letters “a–d” indicate significant differences (*p* < 0.05).

During the storage of fish meat, due to the combined action of microorganisms and endogenous fish enzymes, fish proteins are degraded and amines are produced, resulting in an increase in TVB-N values, and therefore, TVB-N values are an important indicator of the degree of spoilage of aquatic products. Figure 4 shows the effect of repeated freeze–thawing on the change of TVB-N value content of cuttlefish meat. From Figure 8, it can be seen that the rising trend of TVB-N content changes in cuttlefish flesh was relatively stable, and the TVB-N content of cuttlefish flesh was positively correlated with the number of freeze–thaws. The initial TVB-N content of cuttlefish flesh was 5.51 mg/100 g, and the increasing trend of TVB-N content after one freeze–thaw cycle was not obvious. After the third freeze–thaw cycle, it increased to 10.08 mg/100 g and reached 14.18 mg/100 g at the end of the fifth freeze–thaw cycle.

### 3.12. Microstructure

**Figure 9:**
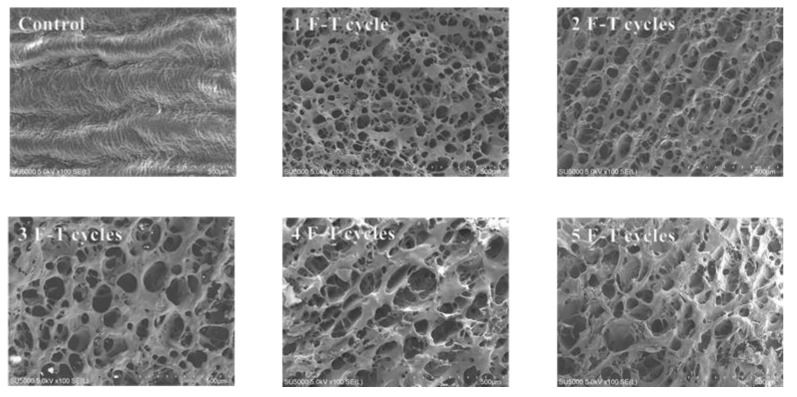
Changes in the microstructure of cuttlefish caused by freeze–thaw cycles under SEM.

Figure 9 is an SEM image of the effect of freeze–thaw cycles on the microstructure of cuttlefish. From the control group, it can be seen that the muscle tissue lines of the non-freeze–thawed samples were compact and clear, well arranged, and without obvious gaps. After freeze–thawing, the muscle fibers underwent significant structural changes, with significant contraction of muscle fibers and an increase in the space between muscle bundles. After two and three freeze–thaw cycles, the cell gaps were significantly enlarged. After four freeze–thaw cycles, the fiber structure of muscle fibers was distorted. After five cycles of freeze–thawing, the muscle fiber borders were blurred, disorganized and collapsed. This is mainly because the free water and part of the bound water in cuttlefish muscle moved from the inside to the outside of myogenic fibers during multiple freeze–thaw cycles, and even moved to the outside of muscle bundles to form larger ice crystals, thus seriously damaging the tissue structure.

## 4. Conclusions

In this study, with the number of freeze–thaws increasing, the process of ice crystal formation, melting and recrystallization in the muscle cells of cuttlefish caused irreversible physical damage to the muscle cells, leading to a decrease in WHC, hardness elasticity and chewiness. In addition, after the freezing and thawing cycles, the protein undergoes oxidative denaturation and the free amino acid content increased, and the content of TBV-N after the fifth freezing and thawing exceeded the standard of first-class freshness. Therefore, in order to keep the good quality of cuttlefish before consumption, the number of freeze–thawing should be less than five. In conclusion, cuttlefish should be kept in cold chain technology during transportation, storage and consumption to prevent repeated freezing and thawing of muscles caused by temperature fluctuations as much as possible, and temperature fluctuations should not be violent to reduce its quality deterioration.

## Figures and Tables

**Table 1:** Color change of cuttlefish flesh that had undergone repeated freeze–thaw cycles.

F-T Cycles	L*	a*	b*
0	65.49 ± 1.13 ^a^	−2.84 ± 0.10 ^a^	−5.64 ± 0.40 ^a^
1	65.65 ± 0.26 ^a^	−2.80 ± 0.27 ^a^	−5.65 ± 1.10 ^a^
2	59.14 ±1.11 ^b^	−3.46 ± 0.24 ^b^	−5.66 ± 0.45 ^a^
3	51.89 ± 1.61 ^c^	−2.35 ± 0.27 ^a^	−7.49 ± 0.36 ^b^
4	57.90 ± 1.01 ^b^	−3.01 ± 0.22 ^a^	−8.10 ± 0.37 ^b^
5	54.02 ± 0.74 ^c^	−3.21 ± 0.41 ^b^	−5.79 ± 1.14 ^a^

The letters “a–c indicate significant differences (*p* < 0.05).

**Table 2:** Texture profile analysis change of cuttlefish flesh that had undergone repeated freeze–thaw cycles.

**F-T Cycles**	0	1	2	3	4	5
Hardness (g)	42,438.83 ± 1628.43 ^a^	38,855.80 ± 591.46 ^b^	35,806.38 ± 237.32 ^b^	28,311.67 ± 612.78 ^c^	17,880.11 ± 51.20 ^d^	12,404.90 ± 1193.63 ^e^
Springiness	0.958 ± 0.00 ^a^	0.922 ± 0.00 ^a^	0.917 ± 0.01 ^a^	0.916 ± 0.03 ^a^	0.794 ± 0.17 ^bc^	0.526 ± 0.03 ^c^
Cohesiveness	0.740 ± 0.01 ^a^	0.738 ± 0.01 ^a^	0.572 ± 0.23 ^ab^	0.399 ± 0.02 ^ab^	0.246 ± 0.06 ^b^	0.25 ± 0.04 ^b^
Chewiness	30,086.68 ± 1682.64 ^a^	27,658.23 ± 3295.74 ^a^	26,425.71 ± 103.67 ^a^	22,171.80 ± 3120.66 ^ab^	15,435.80 ± 3105.86 ^bc^	10,326.34 ± 161.10 ^c^

The letters “a–e” indicate significant differences (*p* < 0.05).

**Table 3:** Free amino acid content change of cuttlefish flesh that had undergone after freeze–thaw cycles.

	0	1	2	3	4	5
ASP	3.84 ± 0.15 ^e^	3.86 ± 0.01 ^e^	7.03 ± 0.11 ^d^	9.69 ± 0.03 ^c^	12.02 ± 0.00 ^a^	11.57 ± 0.07 ^b^
Thr	18.71 ± 1.81 ^d^	26.87 ± 0.09 ^c^	36.74 ± 0.29 ^b^	43.21 ± 0.14 ^a^	35.80 ± 0.08 ^b^	41.94 ± 0.10 ^a^
Ser	19.92 ± 2.14 ^e^	26.82 ± 0.06 ^d^	36.33 ± 0.04 ^b^	30.50 ± 0.30 ^c^	38.10 ± 0.08 ^b^	45.06 ± 0.25 ^a^
Glu	24.21 ± 1.79 ^e^	19.42 ± 0.02 ^f^	37.94 ± 0.35 ^d^	77.81 ± 0.10 ^a^	50.55 ± 0.04 ^c^	61.54 ± 0.11 ^b^
Gly	13.58 ± 1.25 ^e^	14.99 ± 0.05 ^de^	18.94 ± 0.11 ^c^	22.76 ± 0.01 ^b^	16.83 ± 0.01 ^d^	26.24 ± 0.04 ^a^
Ala	34.00 ± 3.84 ^bc^	30.85 ± 0.05 ^c^	45.27 ± 0.28 ^a^	46.98 ± 0.02 ^a^	38.72 ± 0.06 ^b^	49.61 ± 0.13 ^a^
Cys	2.24 ± 0.25 ^d^	3.73 ± 0.03 ^c^	6.05 ± 0.05 ^a^	4.88 ± 0.04 ^b^	2.27 ± 0.03 ^d^	3.44 ± 0.11 ^c^
Val	17.23 ± 1.59 ^d^	16.22 ± 0.03 ^d^	22.60 ± 0.14 ^c^	30.23 ± 0.03 ^b^	30.71 ± 0.02 ^b^	36.66 ± 0.24 ^a^
Met	15.36 ± 1.21 ^e^	23.55 ± 0.05 ^d^	26.45 ± 0.18 ^c^	40.49 ± 0.08 ^a^	29.30 ± 0.12 ^b^	39.90 ± 0.96 ^a^
lle	12.78 ± 0.69 ^d^	9.53 ± 0.03 ^e^	13.20 ± 0.10 ^d^	19.63 ± 0.00 ^c^	26.06 ± 0.15 ^b^	30.86 ± 1.01 ^a^
Leu	28.54 ± 1.39 ^f^	38.41 ± 0.00 ^e^	45.18 ± 0.29 ^d^	71.34 ± 0.02 ^b^	54.57 ± 0.07 ^c^	78.45 ± 0.53 ^a^
Tyr	15.96 ± 0.32 ^d^	27.65 ± 0.03 ^c^	27.88 ± 0.24 ^c^	46.42 ± 0.24 ^a^	35.12 ± 0.16 ^b^	46.88 ± 0.60 ^a^
Phe	20.25 ± 1.42 ^f^	42.59 ± 0.16 ^d^	37.88 ± 0.26 ^e^	69.14 ± 0.25 ^b^	51.63 ± 0.35 ^c^	80.27 ± 0.84 ^a^
Lys	25.00 ± 0.13 ^f^	34.58 ± 0.07 ^e^	46.36 ± 0.33 ^d^	58.69 ± 0.08 ^b^	50.35 ± 0.08 ^c^	66.46 ± 0.24 ^a^
His	7.04 ± 0.25 ^e^	11.15 ± 0.00 ^d^	15.01 ± 0.11 ^c^	17.28 ± 0.02 ^b^	15.63 ± 0.37 ^c^	20.70 ± 0.02 ^a^
Arg	40.01 ± 6.33 ^bc^	20.22 ± 0.05 ^d^	48.69 ± 0.52 ^ab^	50.47 ± 0.01 ^a^	14.33 ± 0.00 ^d^	36.87 ± 0.01 ^c^
Pro	65.30 ± 10.16 ^b^	43.57 ± 0.49 ^b^	54.21 ± 1.76 ^b^	44.58 ± 1.47 ^b^	36.36 ± 1.50 ^b^	136.75 ± 16.00 ^a^
Total	363.95 ± 31.89 ^d^	394.01 ± 0.18 ^d^	525.74 ± 5.07 ^c^	684.09 ± 2.17 ^b^	538.35 ± 0.43 ^c^	813.19 ± 20.26 ^a^

The letters “a–f” indicate significant differences (*p* < 0.05).

## References

[1] Bouletis A.D., Arvanitoyannis I.S., Hadjichristodoulou C., Neofitou C., Parlapani F.F., Gkagtzis D.C. (2016). Quality changes of cuttlefish stored under various atmosphere modifications and vacuum packaging. J. Sci. Food Agric..

[2] Vaz-Pires P., Seixas P., Mota M., Lapa-Guimarães J., Pickova J., Lindo A., Silva T. (2008). Sensory, microbiological, physical and chemical properties of cuttlefish (*Sepia officinalis*) and broadtail shortfin squid (*Illex coindetii*) stored in ice. LWT Food Sci. Technol..

[3] Badiani A., Bonaldo A., Testi S., Rotolo M., Serratore P., Giulini G., Pagliuca G., Gatta P.P. (2013). Good handling practices of the catch: The effect of early icing on the freshness quality of cuttlefish (*Sepia officinalis* L.). Food Control.

[4] Vaz-Pires P., Seixas P. (2006). Development of new quality index method (QIM) schemes for cuttlefish (*Sepia officinalis*) and broadtail shortfin squid (*Illex coindetii*). Food Control.

[5] Leygonie C., Britz T.J., Hoffman L.C. (2012). Impact of freezing and thawing on the quality of meat: Review. Meat Sci..

[6] Wang B., Du X., Kong B., Liu Q., Li F., Pan N., Xia X., Zhang D. (2020). Effect of ultrasound thawing, vacuum thawing, and microwave thawing on gelling properties of protein from porcine longissimus dorsi. Ultrason. Sonochem..

[7] Alonso V., Muela E., Tenas J., Calanche J.B., Roncalés P., Beltrán J.A. (2016). Changes in physicochemical properties and fatty acid composition of pork following long-term frozen storage. Eur. Food Res. Technol..

[8] Yang H., Meng P.P., Wang R., Li P.R., Li P., Wang C.L., Ma L.Z. (2012). Effect of oxidized myofibrils protein subjected to mutiple freeze-thaw cycles on N-nitrosamine formation in in vitro model system (Conference Paper). Adv. Mater. Res..

[9] Cheng S., Wang X., Li R., Yang H., Wang H., Wang H., Tan M. (2019). Influence of multiple freeze-thaw cycles on quality characteristics of beef semimembranous muscle: With emphasis on water status and distribution by LF-NMR and MRI. Meat Sci..

[10] Paarup T., Sanchez J.A., Peláez C., Moral A. (2002). Sensory, chemical and bacteriological changes in vacuum-packed pressurised squid mantle (*Todaropsis eblanae*) stored at 4 °C. Int. J. Food Microbiol..

[11] Qi J., Li C., Chen Y., Gao F., Xu X., Zhou G. (2012). Changes in meat quality of ovine longissimus dorsi muscle in response to repeated freeze and thaw. Meat Sci..

[12] Hu C., Xie J. (2021). The Effect of Multiple Freeze–Thaw Cycles on the Microstructure and Quality of *Trachurus murphyi*. Foods.

[13] Tan M., Wang J., Li P., Xie J. (2020). Storage time prediction of glazed frozen squids during frozen storage at different temperatures based on neural network. Int. J. Food Prop..

[14] Lan W., Sun Y., Chen M., Li H., Ren Z., Lu Z., Xie J. (2021). Effects of pectin combined with plant essential oils on water migration, myofibrillar proteins and muscle tissue enzyme activity of vacuum packaged large yellow croaker (*Pseudosciaena crocea*) during ice storage. Food Packag. Shelf Life.

[15] Tan M., Ye J., Chu Y., Xie J. (2021). The effects of ice crystal on water properties and protein stability of large yellow croaker (*Pseudosciaena crocea*). Int. J. Refrig..

[16] Lan W., Hu X., Sun X., Zhang X., Xie J. (2020). Effect of the number of freeze-thaw cycles number on the quality of Pacific white shrimp (*Litopenaeus vannamei*): An emphasis on moisture migration and microstructure by LF-NMR and SEM. Aquac. Fish..

[17] Lv Y., Chu Y., Zhou P., Mei J., Xie J. (2021). Effects of Different Freezing Methods on Water Distribution, Microstructure and Protein Properties of Cuttlefish during the Frozen Storage. Appl. Sci..

[18] Song Y., Liu L., Shen H., You J., Luo Y. (2011). Effect of sodium alginate-based edible coating containing different anti-oxidants on quality and shelf life of refrigerated bream (*Megalobrama amblycephala*). Food Control.

[19] Tan M., Lin Z., Zu Y., Zhu B., Cheng S. (2018). Effect of multiple freeze-thaw cycles on the quality of instant sea cucumber: Emphatically on water status of by LF-NMR and MRI. Food Res. Int..

[20] Wang X.-Y., Xie J. (2019). Evaluation of water dynamics and protein changes in bigeye tuna *(Thunnus obesus*) during cold storage. LWT.

[21] Wang J., Yu W., Xie J. (2020). Effect of Glazing with Different Materials on the Quality of Tuna during Frozen Storage. Foods.

[22] Chu Y., Cheng H., Yu H., Mei J., Xie J. (2021). Quality enhancement of large yellow croaker (*Pseudosciaena crocea*) during frozen (−18 °C) storage by spiral freezing. CyTA J. Food.

[23] Liu W., Mei J., Xie J. (2021). Effect of locust bean gum-sodium alginate coatings incorporated with daphnetin emulsions on the quality of *Scophthalmus maximus* at refrigerated condition. Int. J. Biol. Macromol..

[24] Zhang M., Li F., Diao X., Kong B., Xia X. (2017). Moisture migration, microstructure damage and protein structure changes in porcine longissimus muscle as influenced by multiple freeze-thaw cycles. Meat Sci..

[25] Wang Z., He Z., Zhang D., Chen X., Li H. (2021). Effect of multiple freeze-thaw cycles on protein and lipid oxidation in rabbit meat. Int. J. Food Sci. Technol..

[26] Nian L., Cao A., Cai L., Ji H., Liu S. (2019). Effect of vacuum impregnation of red sea bream (*Pagrosomus major*) with herring AFP combined with CS@Fe_3_O_4_ nanoparticles during freeze-thaw cycles. Food Chem..

[27] Qiu H., Guo X., Deng X., Guo X., Mao X., Xu C., Zhang J. (2020). The influence of endogenous cathepsin in different subcellular fractions on the quality deterioration of Northern pike (*Esox lucius*) fillets during refrigeration and partial freezing storage. Food Sci. Biotechnol..

[28] Wang Y., Miyazaki R., Saitou S., Hirasaka K., Takeshita S., Tachibana K., Taniyama S. (2018). The effect of ice crystals formations on the flesh quality of frozen horse mackerel (*Trachurus japonicus*). J. Texture Stud..

[29] Cai L., Nian L., Cao A., Zhang Y., Li X. (2020). Effect of Carboxymethyl Chitosan Magnetic Nanoparticles Plus Herring Antifreeze Protein on Conformation and Oxidation of Myofibrillar Protein from Red Sea Bream (*Pagrosomus major*) after Freeze-Thaw Treatment. Food Bioprocess Technol..

[30] Benjakul S., Bauer F. (2000). Physicochemical and enzymatic changes of cod muscle proteins subjected to different freeze-thaw cycles. J. Sci. Food Agric..

[31] Kong C., Wang H., Li D., Zhang Y., Pan J., Zhu B., Luo Y. (2016). Quality changes and predictive models of radial basis function neural networks for brined common carp (*Cyprinus carpio*) fillets during frozen storage. Food Chem..

[32] Li P., Mei J., Xie J. (2021). Chitosan-sodium alginate bioactive coatings containing ε-polylysine combined with high CO_2_ modified atmosphere packaging inhibit myofibril oxidation and degradation of farmed pufferfish (*Takifugu obscurus*) during cold storage. LWT.

[33] Özden Ö. (2005). Changes in amino acid and fatty acid composition during shelf-life of marinated fish. J. Sci. Food Agric..

[34] Baranenko V.K.D., Broyko Y. (2014). Effect of cold treatment on the amino acid composition of veal. Agron. Res..

[35] Gokoglu N., Topuz O.K., Yerlikaya P., Yatmaz H.A., Ucak I. (2018). Effects of Freezing and Frozen Storage on Protein Functionality and Texture of Some Cephalopod Muscles. J. Aquat. Food Prod. Technol..

[36] Shi J., Wang Q., Pan D., Liu T., Jiang M. (2017). Characterization of interactions of simvastatin, pravastatin, fluvastatin, and pitavastatin with bovine serum albumin: Multiple spectroscopic and molecular docking. J. Biomol. Struct. Dyn..

[37] Cai L., Nian L., Zhao G., Zhang Y., Sha L., Li J. (2019). Effect of Herring Antifreeze Protein Combined with Chitosan Magnetic Nanoparticles on Quality Attributes in Red Sea Bream (*Pagrosomus major*). Food Bioprocess Technol..

[38] Xia W., Ma L., Chen X., Li X., Zhang Y. (2018). Physicochemical and structural properties of composite gels prepared with myofibrillar protein and lecithin at various ionic strengths. Food Hydrocoll..

[39] Cao Y., Xiong Y.L. (2015). Chlorogenic acid-mediated gel formation of oxidatively stressed myofibrillar protein. Food Chem..

